# Ecocentrism and Biosphere Life Extension

**DOI:** 10.1007/s11948-022-00404-2

**Published:** 2022-10-26

**Authors:** Karim Jebari, Anders Sandberg

**Affiliations:** 1 Institute for Futures Studies, Stockholm, Sweden; 2 Future of Humanity Institute Oxford Martin School Reuben College University of Oxford, Oxford, UK

**Keywords:** Environmental ethics, Ecocentrism, Anthropocentrism, Biosphere, Long-term futures

## Abstract

The biosphere represents the global sum of all ecosystems. According to a prominent view in environmental ethics, ecocentrism, these ecosystems matter for their own sake, and not only because they contribute to human ends. As such, some ecocentrists are critical of the modern industrial civilization, and a few even argue that an irreversible collapse of the modern industrial civilization would be a good thing. However, taking a longer view and considering the eventual destruction of the biosphere by astronomical processes, we argue that humans, a species with considerable technological know-how and industrial capacity could intervene to extend the lifespan of Earth’s biosphere, perhaps by several billion years. We argue that human civilization, despite its flaws and harmful impacts on many ecosystems, is the biosphere’s best hope of avoiding premature destruction. We argue that proponents of ecocentrism, even those who wholly disregard anthropocentric values, have a strong moral reason preserve the modern industrial civilization, for as long as needed to ensure biosphere survival.

## Introduction

An existential catastrophe is an outcome where the potential of Earth-originating intelligent life is drastically and irreversibly curtailed. Such an outcome could be the result of human extinction, a permanent collapse in human civilization, or a permanent totalitarian world government (Baum et al., 2019; Bostrom & Ćirković, 2008). While many scholars across various disciplines agree that an existential catastrophe would be a negative outcome (Bostrom, 2003; Häggstrom, 2016; Ord, 2020; Scheffler, 2018), their supporting arguments are anthropocentric or focused on human values. For example, Scheffler (2018) argues that human extinction or a permanent collapse of civilization would deprive us of any future that makes our projects worthwhile. Bostrom (2003) argues that an existential catastrophe would drastically reduce the potential amount of human happiness and individual flourishing in the universe. According to perfectionist theories, art, science, and the human experience would also be lost in some existential catastrophes, the value of which cannot be reduced to happiness (Scanlon, 1998). While hedonism and perfectionism are not necessarily anthropocentric, they are often expressed in such terms, especially hedonic theories that distinguish between ‘higher and lower pleasures’ (Mill, 2002). This is also true for those views that welcome human extinction. For example, Benatar (2008) argues that non-existence is better than existence, and that we should not reproduce, even when this would in the long run imply human extinction. From this viewpoint, human non-existence is desirable, because to avoid human suffering matters more than anything else, including non-anthropocentric values.

Yet, not all normative theories are anthropocentric. Authors in the field of environmental ethics argue instead for the need to protect and preserve ecosystems such as coral reefs and mangrove forests, as well as the biosphere as a whole (Brennan & Lo, 2020; Callicott, 1999; Naess, 1973). This view is known as ecocentrism. Human civilization (both modern and pre-modern) poses a considerable threat to various ecosystems (Barnosky et al., 2011; IPBES, 2019), which motivates some proponents of ecocentrism to advocate anti-modernism and primitivism, or the view that human civilization should be materially simplified (Harrison & Tanner, 2011; MacCormack, 2020). By contrast, this article will argue that (1) the biosphere will be destroyed by the increased luminosity of the sun in about a billion years, and (2) that human civilization is currently necessary for extending the lifespan of the biosphere beyond this point. Consequently, there are strong instrumental reasons for ecocentrists to prevent the permanent collapse of modern industrial human civilization. Whereas there are many anthropocentric reasons to prevent such a collapse, we present here an argument that does not assume anthropocentrism.

The article will proceed as follows. First, we describe ecocentrism and its emphasis on holistic systems. We then describe some long-term astronomical threats to the biosphere before describing some potential technological responses. In the final section, we discuss objections, arguing that since human civilization needs to be preserved to prevent the premature (i.e., in one billion years) destruction of the biosphere, this provides ecocentrists with strong reasons to prevent a collapse of civilization.

## Ecocentrism

Ecocentrism is an important normative theory in the field of environmental ethics. Fundamentally, ecocentrism argues that ecosystems matter, not only for the benefits they provide humans and non-human animals with, but for their own sake. In other words, ecocentrism rejects anthropocentrism, or the notion that all value is related to human interests. Ecocentrism is distinct from biocentrism (the view that all organisms, and not only humans, matter) by including ecosystems as holistic entities, including their non-living components (Washington et al., 2017). This is an important aspect of ecocentrism. While ecocentric philosophers believe that individual humans and animals ought to be respected as parts of their respective ecosystems, when the interests of ecosystems are in conflict with the interests of human and non-human animals, some ecocentrists believe that it could be more important to protect the ecosystems. Washington et al. agree with Stan Rowe that:

“Reflecting on the ecological status of all organisms, it [ecocentrism] comprehends the Ecosphere as a Being that transcends in importance any one single species, even the self-named sapient one” (Rowe 1994, quoted in Washington et al., 2017).

One such example has been discussed by Oscar Horta, concerning ongoing efforts at introducing predator species into ecosystems to create an “ecology of fear” (Horta, 2011). Here, it is clear that those advocating the introduction of predators see the interests of plant-eating animals as less important than the flourishing of the ecosystem as a whole.

An ecosystem, in this context, is a community of organisms that interact with each other and the non-living environment as a system. For example, a forest is an ecosystem where many organisms are interconnected and interact with each other and the water, soil, and air in various complex ways. According to some ecocentrists, the value of the forest generates a *prima facie* moral duty on the part of humans to preserve it or promote its goods, be it resilience or biodiversity, as ends in themselves (Callicott, 1999; Curry, 2011; Naess, 1973; Rolston, 1985, 1992). In turn, the *biosphere* is the sum of all ecosystems on Earth. While no natural ecosystems are closed off, the biosphere represents a closed system, at least concerning matter. The biosphere can thus be understood as a distinct object composed of all the objects that, according to ecocentrism, are important.

According to mainstream deontology, duties are directed against right-holders, such as persons or individuals with interests. This view has been challenged in environmental ethics in general and in ecocentric ethics in particular, as ecosystems and other objects of value are often not sentient beings, as Sandler (2010) argues. Philosophers concerned about duties in environmental ethics, often conceptualize these duties as general principles of proper conduct. The duty to preserve ecosystems can in this view be seen as respect for those entities, rather than as a rights-based duty. This is one aspect where environmental ethics differs from other forms of normative theory (Taylor, 1981).

Other philosophers argue that non-sentient entities such as species and ecosystems can be seen as moral patients, even when they lack what we typically think of as interests. For example, Holmes Rolston III (1985) argues that holistic entities such as species and ecosystems, have interests that are distinct from but analogous to those of humans and animals, and that killing a species is the ‘super killing’ of a whole pattern of life. This line of reasoning could be extended to the entire tree of life. If pruning a branch on the tree (i.e., killing a species) of life is a “super killing”, then killing the entire tree would arguably constitute a “total killing”. This notion has certain similarities with the idea of *ultimate harm*, or the destruction of all value in a domain (Persson & Savulescu, 2008).

While some authors like Hiller (2013) endorse a form of ecocentric consequentialism, this view is unusual among ecocentrists (Hiller, 2017). However, this view should not be conflated with the view that we ought to maximize the objects that are value-bearers. This view is even more unusual among ecocentrists, although it has been defended by Tonn (1999), and more recently by Owe (2022) who have argued that we have an obligation to seed the universe with life. Maher and Baum (2013) also argue that ecocentric value could be vastly increased by interstellar expansion, which suggests an aggregative view of ecocentric value (i.e., the view that “more is more”). However, most ecocentric philosophers argue that we have a duty to *preserve and protect* (rather than promote) ecosystems, especially those that make up our planet’s biosphere. Similarly, Scanlon (1998) argues that some values ought to be protected and not maximized.

The implication of rejecting maximizing consequentialist ecocentrism is that while humanity may, in the distant future, choose to radically increase the number of ecosystems in the universe, this is not necessarily something that would count in its favor. From a mainstream (i.e., non-maximizing) ecocentric perspective, what matters is protecting the ecosystems that exist, not maximizing their numbers. In other words, the creation of life on other planets cannot compensate for the destruction of life on this one. This is especially true on the view defended by Holmes Rolston III (2012) according to which the value of ecosystems in parts depends on their particular evolutionary history, a history that cannot be continued unless this particular biosphere is preserved.^1^

Human civilization and its relentless economic activity represent a considerable near-future threat to various ecosystems, according to the 2019 Global Assessment Report on Biodiversity and Ecosystem Services (Barnosky et al., 2011; IPBES, 2019). From an ecocentric perspective, then, it can be argued that modern civilization and industry are major sources of value destruction (Best, 2014). Many environmental ethicists and ecocentrists are also value pluralists and therefore recognize at least some anthropocentric values, while others simply deny that we can separate “nature” from the “human”, as the view that “nature must be protected from humans” implies (Morton, 2007). However, modern ecocentrists have been very critical of modern industrial civilization, and its immediate harms on the biosphere, while long term considerations have rarely been discussed. The implication of such views has sometimes been that modern civilization is considered harmful or undesirable (Harrison & Tanner, 2011; MacCormack, 2020).

We argue, in line with ‘distant environmental ethics’ (Owe, 2022; Tonn, 2002), that from a long-term perspective, human civilization and its technological tools currently represent the best hope for the long-term survival of life on Earth, and perhaps in this galaxy. Specifically, the biosphere is likely to crash in about a billion years due to the increased luminosity of the Sun. From an ecocentric perspective, this is a significant concern since it implies *ultimate harm* (Persson & Savulescu, 2008), that is, the loss of all value structures and the irreversible loss of all potential to produce further value. Consequently, we argue that there are strong ecocentric reasons to preserve modern civilization.

## Long-Term Threats to the Biosphere

Over the long term, the Earth and the Sun are irreversibly changing. The Earth’s core will cool down, leading to a weakened continental cycle, and the Sun’s luminosity will gradually increase, eventually transforming the Sun into a red giant. These trends will impact the biosphere and eventually cause its destruction. This is different from other past mass extinction events, as these were consistent with the possibility of life in the future. Here, the long-term changes under consideration will *permanently* end the Earth’s suitability for life.

### Breakdowns of Biogeosphere Homeostasis

The carbonate–silicate cycle is an important aspect of the Earth’s thermal regulation. At present, carbon dioxide (CO_2_) provides a greenhouse effect that keeps Earth warm enough for water to be liquid at the surface, maintains temperature homeostasis, and makes photosynthesis possible. In the long term, this homeostasis will break down and likely produce a state in which normal photosynthesis is impossible, ending the biosphere as we know it.

As part of this cycle, carbon dioxide is removed from the atmosphere by the weathering of silicate rocks in warm and wetland environments. Dissolved carbonates and silicates eventually precipitate on the sea floor, forming sedimentary rocks. These sediments are subducted by continental drift; at the higher subterranean temperature, they recombine to produce the original silicates and CO_2_. The latter is released through volcanism, maintaining a higher concentration in the atmosphere than the weathering chemical equilibrium would entail (Pierrehumbert, 2010). However, this cycle is not necessarily a closed loop, since the amount of CO_2_ that flows back into the atmosphere depends on the rate of subduction and volcanism. Were these to slow down or cease, CO_2_ levels would fall as weathering would irreversibly remove the gas.This cycle also serves to regulate surface temperature, removing more CO_2_ at high temperatures and allowing it to build up during cold periods (Walker et al., 1981). While inorganic weathering is a slow process that currently accounts for far less carbon flow (about 0.2 gigatonnes per year) than the biosphere and anthropic processes (about 120 and 7 gigatonnes per year, respectively), it is linked to vast reservoirs that are at least 20,000 times larger than the combined atmospheric and biospheric carbon (Martin, 2017). This allows the carbonate–silicate cycle to regulate not only the temperature but, to some extent, the mass of the biosphere since it is in part constrained by the amount of CO_2_ available for photosynthesis.

The loss of radioisotopes and accretion heat in the Earth’s core will eventually end the recycling of CO_2_ through tectonic activity. In the absence of human intervention, this will lead to a significant loss of greenhouse effect and plant viability, presumably leading to a cold and barren environment in a few billion years.

Conversely, the Sun’s luminosity is also slowly increasing; the effects of this process will likely counteract the cooling effect of reduced tectonic activity, but also speed up the weathering effect. As hydrogen atoms are fused, the proportion of helium atoms in the Sun’s core gradually increases and the core itself becomes denser and hotter, moving hydrogen fusion outwards. At present, this process is increasing the Sun’s luminosity by around 1 per cent every 100 million years and will have a significant warming impact on the biosphere, even in the absence of CO_2_, which is not the only greenhouse gas. In particular, water vapor is a major greenhouse gas and hence a sufficiently hot and humid environment would tend to become hotter, since warm air accelerates evaporation. In certain scenarios, this produces drastically hotter surface temperatures, also known as a ‘runaway greenhouse’ (Goldblatt & Watson, 2012). In this runaway greenhouse scenario, a warmer and hence wetter atmosphere heats the planet up to temperatures that seem inconsistent with complex life, and ultimately up to a point at which the atmosphere becomes steam-dominated and the seas disappear. Moreover, as moisture and heat push water vapor into the upper atmosphere, UV radiation dissociates oxygen and hydrogen from the water molecules. The resulting free hydrogen molecules—which are very fast and thus can easily escape the Earth’s gravity—will be stripped from the atmosphere, eventually drying out the surface completely; this is somewhat confusingly known as the ‘moist greenhouse’ scenario (Kasting, 1988). If the moist greenhouse has not sterilized the planet by this point, the absence of water will make the biosphere even more difficult to sustain. Eventually, sufficient heat may bake CO_2_ out of carbonate rocks, further boosting the greenhouse effect and making Earth Venus-like. At this point, no lifeform, no matter how simple, seems remotely possible.

The process by which increased solar luminosity will lead to a moist or runaway greenhouse (and ultimately the destruction of the biosphere) interacts with the carbonate–silicate cycle in two primary ways. First, warmer temperatures will interrupt the homeostasis of the cycle, increase weathering, and speed up the process of CO_2_ removal. Second, increased solar luminosity will compensate for the reduced greenhouse effect from the lost CO_2_ for a limited time; eventually, despite the lack of CO_2_, the planet will continue to heat up. While the warming induced by increased solar luminosity will for some time be offset by increased weathering of CO_2_, this will also reduce the potential for photosynthesis and eventually lead to a runaway greenhouse state with surface temperatures exceeding 100 °C. Even if some simple life forms may survive this, the Earth’s biosphere will ultimately be sterilized without any surface water.

Aside from these inevitable changes, other biosphere-ending disasters may also occur. Over the next billion years, we should expect a certain amount of large asteroid or comet impacts and supernovas that are severe enough to cause mass extinctions (Beech, 2011). Sufficiently large impacts to wipe out the entire biosphere are less likely, but not impossible (Sloan et al., 2017). Likewise, there is a small risk (less than 1 per cent) of orbit disruption through close stellar encounters (Malmberg et al., 2011) or orbital instability in the inner solar system (Laskar & Gastineau, 2009). We will not deal with these risks here, other than noting that interventions to prevent some biosphere-ending disasters are possible and raise similar ethical issues.^2^

### Estimates of Remaining Biosphere Lifespan

**Fig. 1:**
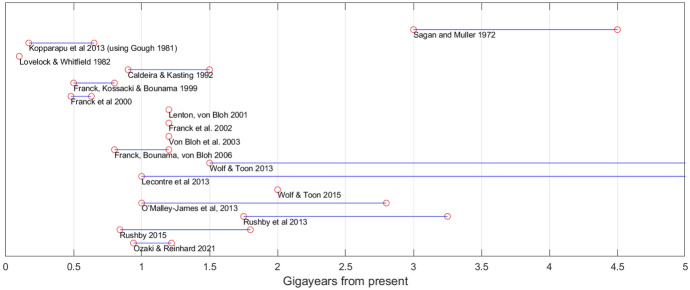
Published time estimates for the end of Earth’s biosphere

One of the first scientific estimates^3^ of future biosphere lifespan was made by Sagan and Mullen (1972), who predicted a runaway greenhouse state in 3–4.5 Gyr (billions of years) due to increasing water vapor as the planet heats up due to rising solar input. Since then, increasingly sophisticated models of interactions between the biosphere, geosphere, atmosphere, and solar input have been used to estimate the limits of the future biosphere. Lovelock and Whitfield (1982) estimated that CO_2_ levels will fall below the critical level for C3 photosynthesis (the most common type) in only 100 Myr (millions of years). Caldeira and Kasting (1992) re-examined the problem with a more elaborate model (and focusing on C4 photosynthesis), positing a lifespan of 0.9–1.5 Gyr. Franck et al. (1999) extended this model, finding that photosynthesis breaks down in 0.5–0.8 Gyr and surface temperatures reach 100 °C in 1–1.5 Gyr. Other models and assumptions have produced estimates of the remaining biosphere lifespan of 0.48–0.63 Gyr (Franck et al., 2000), 1.2 Gyr (Franck et al., 2002; Lenton & von Bloh, 2001; von Bloh et al., 2003), 0.84 Gyr (Rushby, 2015) and 1.08 Gyr (Ozaki & Reinhard, 2021) (Fig. 1 and Table 1).

**Table 1:** Published time estimates for the end of Earth’s biosphere

Source	Time estimate	Cause
Sagan and Mullen (1972)	3–4.5 Gyr	Runaway greenhouse
Lovelock and Whitfield (1982)	100 Myr	CO_2_ depletion
Caldeira and Kasting (1992)	0.9–1.5 Gyr	CO_2_ depletion
Franck et al. (1999)	0.5–0.8 Gyr 1–1.5 Gyr	CO_2_ depletion Surface temperatures 100 °C
Franck et al. (2000)	0.48–0.63 Gyr	CO_2_ depletion
Franck et al. (2002)	1.2 Gyr	CO_2_ depletion
Lenton and von Bloh (2001)	1.2 Gyr	Overheating
von Bloh et al. (2003)	1.2 Gyr	Overheating
Franck et al. (2006)	0.8–1.6 Gyr	Overheating, gradually affecting multicellular, eukaryotic and prokaryotic life
O’Malley-James et al. (2012)	1.0 Gyr 2.8 Gyr	Moist greenhouse Overheating
Rushby et al. (2013)	1.75–3.25 Gyr	Overheating
Leconte et al. (2013)	1.0–5.0 Gyr	Runaway greenhouse
Kopparapu et al. (2013) (using solar model from Gough, 1981)	0.17 0.65 Gyr	Moist greenhouse Overheating
Rushby (2015)	0.84 Gyr 1.8 Gyr	CO_2_ depletion Runaway greenhouse
Wolf and Toon (2014)	1.5–5.0 Gyr	
Wolf and Toon (2015)	2.0 Gyr	Moist greenhouse
Ozaki and Reinhard (2021)	1.08 Gyr	CO_2_ depletion and deoxygenation (followed by moist greenhouse)

When these processes will ultimately destroy the last organism on Earth depends on assumptions about the heat tolerance of different kinds of life. Franck et al. (2006) found future extinction times of 0.8–1.2 Gyr for multicellular life, 1.3–1.5 Gyr for eukaryotes and 1.6 Gyr for prokaryotes using the same basic model. O’Malley-James et al. propose that while a moist greenhouse may set in 1 Gyr from now, small refugia at high latitudes and altitudes may sustain extremophiles up to 2.8 Gyr (O’Malley-James et al., 2012).^4^ On the contrary, it may be objected that the biosphere has proved remarkably resilient over the most recent billions of years. It has persisted in the face of extreme and violent events such as abrupt climatic changes, massive volcanism and asteroid collisions (O’Malley-James et al., 2012). Moreover, the events described here will take place over a timespan that allows for the evolution of adaptive traits. Even if no existing species would survive a biosphere without CO_2_ or a moist greenhouse, such species could conceivably evolve. Consider the first (known) mass extinction event: the Great Oxygenation Event. Although very few species had evolved tolerance for oxygen (a highly reactive gas), life evolved and adapted nonetheless (Margulis & Sagan, 1997).While it is true that life is very resilient, especially in simple lifeforms, those ecosystems that can survive will be very simple and biodiversity will be very low. While some parts of the biosphere could conceivably survive for hundreds of millions of years after the runaway phenomena described above, no multicellular life is likely to ever emerge again. While some value-bearing systems would persist for a while, complex valuable ecosystems would not. Eventually, without intervention, even the most resilient organisms would disappear. Note that the outcome of these processes is not merely an ‘extinction event’ nor even a ‘mass extinction event’. Rather, we refer to the outcome as *sterilization*. On the one hand, extinction is where a species or a phylogenetic group dies out. On the other hand, sterilization is the permanent elimination of all lifeforms on a planet. Whereas extinction may be a natural part of the history of life, sterilization is the ending of that history. Whether sterilization is ‘natural’ or not depends on the exact definition of ‘natural’ in this context. Mass extinction events in a biosphere add to the richness of its history and enable new forms of life to emerge; consider, for example, the evolutionary radiations of mammals and birds after the Cretaceous-Triassic mass extinction. However, biospheres are causally isolated from each other^5^: one biosphere’s death does not benefit any other biosphere.

In summary, modern biosphere/geosphere models tend to suggest a minimum of 800 Myr of remaining lifespan and 1.2 Gyr as a plausible median. Crucially, *all* models imply that the biosphere’s future is shorter than its past. While future evolutionary adaptations will likely help life to persist where current life would perish, there are limits to what natural evolution can achieve.

## Potential Responses

Technological interventions could postpone the biosphere’s demise in several ways. Here, we discuss their feasibility given the assumption that human civilization survives over the relevant periods. These are some suggestions found in the literature and should not be seen as an exhaustive list. Whether or not these suggestions are feasible in the future remains to be seen. The basic point is that something can be done at a moderate cost to a future civilization with adequate resources and technological tools.

### Moving the Earth to a More Remote Orbit

While extreme amounts of momentum are required to change planetary orbits appreciably, the slowness of solar brightening also means the methods do not have to be particularly fast. Any orbit-change scheme will still by necessity involve managing energy on a scale comparable to the climate. McInnes (2002) suggests a gravitational tug approach to move the Earth, while Brin (2014) proposes using a solar-powered electrodynamic tether on the far side of the moon. One scenario consists in using streams of asteroids to transfer momentum between Jupiter and Earth using gravity assist maneuvers (Korycansky et al., 2001). While some of these solutions could be dangerous, there is ample time to develop technologies and study the problem over the coming millennia.

### Tuning Atmospheric Chemistry

This would involve efforts either to reduce the greenhouse effect (adding up to 2.3 Gyr) (Li et al., 2009) or to counteract CO_2_ depletion by artificially releasing bound carbonates.^6^ As the current climate change problem shows, human civilization is already technologically capable of climate-affecting atmospheric chemistry.

### Solar Input Control

Sagan and Muller proposed aerosol geoengineering in 1972 but assumed it would be hard to achieve even with very advanced technology. From our current perspective, geoengineering may appear more feasible (Goldblatt & Watson, 2012). However, aerosols need to be replenished often and cannot realistically handle insolation beyond a certain critical level without becoming opaque, upsetting the light and temperature balance. Artificial rings around Earth have been suggested to reduce global warming (Bewick et al., 2013; Mautner, 1991; Pearson et al., 2006) with typical estimated insolation changes in the order of 1.7 per cent. While this may prove enough to deal with current anthropogenic climate change, it will be difficult to scale up to the degree needed to handle increasing solar luminosity.

Solar shades located between the Earth and the Sun have also been suggested as a remedy for current climate change (Early, 1989; Mautner, 1991; McInnes, 2010; Teller et al., 1996). Angel (2006) suggests swarms of thin film refractive discs, while other proposals involve an occulting disc or swarm (Hudson, 1991; McInnes, 2002, 2006). In all these cases, active control is needed for station-keeping, or the system will slowly drift out of alignment (a problem already solved for current satellites). These proposals typically suggest that the cost is within near-future reach for current human civilization (a few per cent of global GDP at most). Using Gough’s (1981) solar luminosity formula posits that, over the next Gyr, insolation will increase by 9.6 per cent. This is within the range of what these solar shades could address without too radical an engineering challenge. The 21 per cent and 35 per cent increases in luminosity over the following two billion years, respectively, may be more challenging. A workable solar shade would need to both slowly increase in radius (since the solar radius is increasing) and opacity (to maintain the same effective insolation). In principle, it would appear that this system could function up until the Sun starts to affect the Earth more directly in 7.4 Gyr.^7^

The main drawback of this strategy is that the solar shade would need to be continually maintained. Self-replicating technology could autonomously replenish the swarm (for example, by mining asteroids and sending new components to Earth using solar sails when the shade is too thin). The total mass involved does not have to be enormous. An Earth-sized total shade would mass about 2 billion tonnes, or about 31 years of current annual aluminum production. The required growth rate is modest. For the next billion years, about one 100×100-m section would need to be added per year, weighing 15 kg (at the time of writing, the Planetary Society LightSail-2 craft that orbits the Earth has an area of 32 m^2^ and weighs 5 kg).

## Discussion

Based on the above, it would seem that it is physically *possible* for an advanced civilization to reduce insolation or other factors in such a way that the biosphere could persist for at least an additional billion years. Although it is not *at present* technically feasible to do so, the aforementioned techniques for solar shades are straightforward extrapolations of current satellite station-keeping methods and solar sails. The most speculative part is automatic mining and manufacturing, but it does not appear implausible that such systems could be built over the coming centuries or millennia given current work on in-situ resource utilization in space. Even based on conservative assumptions, it seems feasible that a species with advanced technology could, through various engineering projects, extend the viability of Earth’s biosphere for at least one billion years. We have transitioned from pre-agricultural technology to the Anthropocene in a mere 10,000 years, and it is hard to imagine what capabilities would be available were human civilization to survive for another 10,000 years, or even 100,000 years. The same is true for our understanding of the biosphere and the factors that affect its survival. Granted, the methods outlined in this paper are likely to be superseded by something more workable, but this does not mean that protecting the biosphere should not be done in the here and now.

It should be noted that these interventions are not a *restoration* but a *preservation* of the biosphere. Building a solar shield does not *create* a biosphere; it is *preventing* the biosphere’s premature destruction. In other words, installing a solar shield is not equivalent to replanting a forest or recreating a marshland. Rather, it is the equivalent of building a levee to prevent saline water from the sea from destroying the ecosystem in a freshwater lake. It is important to distinguish between restoration and preservation here because some prominent authors have argued against the idea that natural ecosystems can and should be restored and that this restoration would reverse the environmental harm caused. For example, Elliot (1982) argues that the value of an object depends partly on its origins or how it came about. Similarly, Katz (1996) states that the attitude that constitutes the restorative ideal is such that restoration confers no *natural* value at all, only *instrumental* value. Katz and Elliot could therefore argue that preservation would *also* destroy the value of the protected ecosystem, but it does not follow from the claim that restored ecosystems lack value. Thus, even if we accepted the argument against restoration, it would not apply to the moral value of protecting the biosphere.

A similar but more recent discussion concerns whether humans should intervene to protect wildlife against disasters such as forest fires. Here, interventionists argue that the natural world and human civilization are now so intertwined that what happens in the natural world becomes our concern (McCumber & King, 2020). Other interventionists have argued that large-scale interventions are justified because it is our primary duty to prevent harm (Johannsen, 2020). These pro-intervention arguments apply not only to the current era (the Anthropocene) but also to the far future. It is a fact that humanity has irreversibly changed the history of life on this planet, and that we will continue to do so for as long as we exist. On the other hand, it is equally important to note that the survival of human civilization is currently *necessary* for the life extension of the biosphere to happen. While we may develop (at some point in the future) technology advanced enough to autonomously preserve the biosphere in our absence, such technology is far beyond our current grasp. Thus, to be of use to the biosphere, human civilization must survive at least until the necessary technology has been developed.

One possibility is that if humanity goes extinct, a future species with similar ability to use technology will evolve and extend the biosphere lifespan. While certainly possible, the time remaining is finite, and the appearance of species able to use technology appears to be rare and random.^8^ Reliance on future species thus represents a major gamble with the biosphere. And we have no reason to believe that any hypothetical future species would be more inclined to avoid environmental harm or to extend the lifespan of the biosphere in the future.

To clarify, we are not suggesting that it is possible to *prevent* the eventual sterilization of the biosphere. Even if it would be possible to reduce the amount of radiation to offset the increase in luminosity over the next billion years, the Sun will eventually become a red giant and swallow the Earth. And even if the Earth could be moved to another orbit, the fusion in the Sun’s core will eventually cease, and the Sun will become a white dwarf. Sooner or later, then, the biosphere will be sterilized, and humanity (or, rather, post-humanity) will no longer be able to prolong the lifespan of the biosphere. What we are suggesting is that the natural sterilization of the biosphere could be delayed. If the finitude of the biosphere contributes to its value, we can rest assured that this source of value is not threatened by human activity.

In summary:

1. According to ecocentric ethics, the biosphere is what matters.
2. The continued existence of human industrial civilization is currently necessary to prevent the premature destruction of the biosphere.

So,

(3) Assuming ecocentric ethics, we have strong reasons to ensure the continued existence of human industrial civilization.

## Objections

*Anthropogenic destruction of the biosphere* Human industrial civilization may cause the premature destruction of the biosphere. Human industrial civilization has caused much harm to the biosphere, even with today’s limited technology. There is a risk that future technologies could destroy the biosphere long before the geophysical processes outlined in this article. While it is possible that human civilization could save the biosphere, it is also possible that it could destroy the biosphere. When we can’t assign probabilities to two possible outcomes that are symmetrical and opposite in value, we should suspend judgement about which outcome is preferable.

Response: The outcomes are not symmetrical. In the absence of technological intervention, the destruction of the biosphere is almost certain. By contrast, while the destruction of the biosphere with anthropogenic means is certainly possible in the long-term future, it would require much more effort and resources than extending the lifespan of the biosphere. The biosphere is not fragile and has withstood several massive chocks. Not even the most apocalyptic nuclear winter scenarios (see Toon et al., 2014) in the literature or the most pessimistic scenario suggested by the IPCC (RCP 8.5, see Newbold, 2018) would suffice to destroy the biosphere. Taking this into consideration, we believe that it is far more likely that future humans (or posthumans) decide to extend the lifespan of the biosphere than they are to destroy the biosphere prematurely. However, this conjecture cannot be proven. Under the assumption that human civilization is more likely to destroy the biosphere than to extend its lifespan, there may indeed be strong instrumental reasons to prefer the irreversible collapse of human civilization.

It is possible that a future non-human civilization may intervene to extend the lifespan of the biosphere in the event of human extinction. But the probability of such a civilization emerging and surviving for long enough is unknown. While it is possible that human civilization destroys or fails to prevent the premature destruction of the biosphere, it is presently the only object that can prevent premature destruction. Here is an analogy.

A man suffers from a slow growing but lethal cancer. Currently, chemotherapy is necessary for his survival. The chemotherapy may not cure the disease, and it may kill the man. Moreover, there is an unknown probability that some treatment in the future may be just as effective. Yet, chemotherapy is presently necessary for the man’s survival. While it may be rash to begin treatment immediately, before it is strictly necessary, it would also be rash to eliminate the option to use chemotherapy entirely. Making sure that human industrial civilization persists would be like keeping chemotherapy as an option.

*Hubris* Humanity has destroyed much value in its hubristic endeavors. Our interactions with the biosphere should avoid hubris, ambitions of ‘playing God’ and efforts to subject the biosphere to our control. To suggest that we ought to preserve the biosphere is an extreme form of the same hubristic attitude and as such is a dangerous endeavor.

Response: If we understand ecocentrism as a theory of how to value certain entities, such as ecosystems, rather than as a more general theory of human virtues, then it is not contrary to ecocentrism to have ambitions that could be described as hubristic. Whether hubris is good or bad is irrelevant from the perspective of the biosphere. Hubris, arrogance, and delusions of godhood are human attitudes and mental states. These are good or bad, depending on which anthropocentric notion of which human vices and virtues we should avoid. However, unless ecocentrism is understood as a theory of human virtues, it is silent on the intrinsic value of different human attitudes. Of course, there is a prudential reason to be wary of playing God: grand interventions can go wrong and cause commensurate damage. Clearly, any attempt at extending the lifespan of the biosphere requires extreme care and caution (especially if it is irreversible, for example, by ceding control over to pre-programmed robots). The high stakes imply that having humans deliberate over or continually monitor the relevant processes for extensive periods is desirable. Starting too early may be a serious mistake, but then humanity needs to survive long enough to start at an appropriate time.

*Animal suffering* Many animals suffer greatly in the biosphere: disease, predation, injury, and starvation are common (Horta, 2015). Extending the lifespan of the biosphere would increase this suffering and is hence undesirable.

Response: If one accepts the objection at full strength, then the conclusion would seem to be that interventions to shorten the lifespan of at least a complex animal-hosting biosphere would be favorable (Schopenhauer, 1958). Others have argued that nature selects for ‘beneficial pain’, and without it there could not be as much value, so one should accept the status quo (Rolston, 1992), in which case the objection loses power. However, a moderate position (still rejecting anthropocentrism) would be that humans have duties of assistance towards animals suffering in the wild (Moen, 2016)—a further reason for human civilization to persist. Note also that while some ecocentrists acknowledge the importance of animal suffering, most argue that it is less important than the continued existence of ecosystems. Indeed, the interests of animals and what benefits an ecosystem’s resilience may sometimes come into conflict in other contexts. For example, it has been suggested that ‘purging’ harmful genes from an inbred population of wolves could make the overall population, and by extension the ecosystem of which the population is part, better off (Liberg, 2005).

*Discounting*^9^ Some ecocentrists might argue that we should not value ecosystems in the distant future of hundreds of millions to billions of years from now in the same way that we should value the near-term future of tens to hundreds of years from now.

Response: The idea of discounting is plausible in some contexts, especially with regards to economic investments. It might for example be better to delay making renovations to your house for a year, if the costs of delay increase less than the costs of the interest on the same sum if it was saved or invested elsewhere. Some economists, most famously William D. Nordhaus argues that we should not only consider the interest rate, but also that we should also include “time discounting” (Nordhaus, 1997). Time discounting is what a person may indulge in when postponing a visit to the dentist, as it involves giving less weight to future well-being. Many philosophers have been very critical to this idea, as it seems to amount to a disregard for objects and individuals depending on when they happen to exist. Just as an object’s location in space is normatively irrelevant, so is an object’s location in time (Caney, 2014, pp. 323–327; Parfit, 1984, pp. 480–486; Rawls, 1999, pp. 259–262).

## Conclusions

A permanent global collapse of human industrial civilization would be a moral disaster according to several normative theories. Thus far, this conclusion has mostly been supported by anthropocentric arguments. However, if human industrial civilization is currently necessary to avoid premature sterilization, then this would imply a strong *instrumental reason* to avoid this outcome, according to ecocentrism. Depending on how much importance is assigned to ecocentric values relative to anthropocentric values, these considerations may override anthropocentric or biocentric reasons for extinction (Benatar, 2008). Even if we believe that the persistence of humanity is bad for humans, the fact that only humanity could protect the biosphere may prove more important. In this case, the instrumental value of continued existence would be greater than the negative intrinsic value of human suffering.^10^

Some authors have argued that a permanent collapse of human civilization would be very bad (Baum et al., 2019; Häggström, 2016; Ord, 2020). These arguments have often assumed an anthropocentric outlook. Ecocentric views have been more ambivalent towards the value of human civilization, sometimes even welcoming its destruction. We have argued here that proponents of ecocentrism have strong normative reasons to prevent this outcome as long as humanity remains the only species able to develop the capabilities of preventing premature destruction of the biosphere.

The timescale of human civilization is vanishingly short when compared to the timescale of the biosphere. But just as it is possible to do harm that has consequences far beyond the present, it may be possible to do good that stretches eons into the future.

## Footnotes

1 We are grateful to an anonymous reviewer for pointing this out.

2 Most straightforward is deflecting impactors. Protection against supernovae and gamma ray bursts has been discussed in Ćirković and Vukotić (2016).

3 The *first* estimate was made by Georges-Louis Leclerc, Comte de Buffon in 1778. Arguing that the Earth was losing heat from an initially hot state and using experiments with heated metal balls to establish scaling relations, he estimated a finite future lifespan of the biosphere before it would freeze to about 93,000 years. He also suggested that this end could be delayed somewhat by increasing the number of warm-blooded animals. While wrong about nearly all details, he was the first to use a scientific approach to consider Earth’s habitability in the future (Leclerc, 2018).

4 The biosphere has an upper lifespan set by the Sun’s red giant phase, peaking in 7.59 billion years. At this point, its radius expands beyond Earth’s current orbit. Whether the planet is absorbed by the Sun or narrowly escapes is uncertain given current knowledge, but it is certain that Earth can no longer sustain organic life by this point (Schröder & Connon Smith, 2008).

5 Hypothetically, there may be panspermia processes in which bacterial spores launched (due to asteroid impacts) on one planet seed other planets with life. If the emergent biospheres are valuable, or this natural process is itself valuable, then the disappearance of a biosphere weakens the emergence of value. In a fecund galaxy filled with biospheres, this may make the loss of one only a minor loss. However, were one to accept this argument, the ecocentric case for protecting Earth from human activity is significantly weakened.

6 Current weathering rates have been estimated to be much smaller than current human emissions. A future endeavour to maintain CO_2_ levels powered by solar energy is hence likely to be feasible even with higher weathering rates.

7 At this point, the expanding Sun will potentially catch up with the Earth’s orbit (see Schröder & Connon Smith, 2008).

8 While estimating the rate of intelligent species emergence is highly nontrivial, it is possible to make probabilistic arguments that take observer selection effects into account (Snyder-Beattie et al., 2021). A consequence of this model is that humans should expect to be roughly in the middle of the time between the earliest an intelligent species could emerge (the previous ‘hard step’ in the model) and the end of the biosphere, and that the probability of another subsequent intelligent species emerging thereafter is less than half of humanity’s probability, which being a hard step in the model is very small.

9 We are thankful to the comment from an anonymous reviewer for proposing this objection.

10 While Benatar finds suffering bad for all beings, he is only advocating human extinction. Others have taken the antinatalist position to its logical conclusion: that it is bad for any sentient being to come into existence and hence humanity should help put the biosphere out of its misery. While Schopenhauer may today be the most famous proponent of this view (‘nature has to expect its salvation from man who is at the same time priest and sacrifice’; 1958), von Hartmann (2014) has explicitly argued that to stop perpetuating the misery of existence, humanity needs to not just wipe out Earth’s biosphere but all other biospheres. Such ecocentric antinatalism is diametrically opposed to the account in this paper, although it would likely agree with us on the instrumental value of human civilisation to attain a good outcome.

## References

[CR1] Angel R (2006). Feasibility of cooling the earth with a cloud of small spacecraft near the inner Lagrange point (L1). Proceedings of the National Academy of Sciences of the United States of America.

[CR2] Barnosky AD, Matzke N, Tomiya S, Wogan GOU, Swartz B, Quental TB, Marshall C, McGuire JL, Lindsey EL, Maguire KC, Mersey B, Ferrer EA (2011). Has the earth’s sixth mass extinction already arrived?. Nature.

[CR3] Baum SD, Armstrong S, Ekenstedt T, Häggström O, Hanson R, Kuhlemann K, Maas MM, Miller JD, Salmela M, Sandberg A, Sotala K, Torres P, Turchin A, Yampolskiy RV (2019). Long-term trajectories of human civilization. Foresight.

[CR4] Beech M (2011). The past, present and future supernova threat to Earth’s biosphere. Astrophysics and Space Science.

[CR5] Benatar D (2008). Better never to have been: The harm of coming into existence.

[CR6] Best S (2014). The politics of total liberation: Revolution for the 21st century.

[CR7] Bewick R, Lücking C, Colombo C, Sanchez JP, McInnes CR (2013). Heliotropic dust rings for Earth climate engineering. Advances in Space Research.

[CR8] Bostrom N (2003). Astronomical waste: The opportunity cost of delayed technological development. Utilitas.

[CR9] Bostrom N, Ćirković MM (2008). Global catastrophic risks.

[CR10] Brennan, A., & Lo, Y.-S. (2020). Environmental ethics. In Edwad N. Zalta (Ed.), *The Stanford encyclopedia of philosophy* (Winter 2020 Edition). https://plato.stanford.edu/archives/win2020/entries/ethics-environmental/.

[CR11] Brin, D. (2014). *Let’s lift the E**arth!* Contrary Brin. Retrieved October 23, 2020, from http://davidbrin.blogspot.com/2014/11/lets-lift-earth.html.

[CR12] Caldeira K, Kasting JF (1992). The life span of the biosphere revisited. Nature.

[CR13] Callicott JB (1999). Beyond the land ethic: More essays in environmental philosophy.

[CR14] Caney S (2014). Climate change, intergenerational equity and the social discount rate. Politics, Philosophy & Economics.

[CR15] Ćirković MM, Vukotić B (2016). Long-term prospects: Mitigation of supernova and gamma-ray burst threat to intelligent beings. Acta Astronautica.

[CR16] Curry P (2011). Ecological ethics. An introduction.

[CR17] Early JT (1989). Space-based solar shield to offset greenhouse effect. Journal of the British Interplanetary Society.

[CR18] Elliot R (1982). Faking nature. Inquiry.

[CR19] Franck S, Block A, von Bloh W, Bounama C, Schellnhuber HJ, Svirezhev Y (2000). Reduction of biosphere life span as a consequence of geodynamics. Tellus B.

[CR20] Franck S, Bounama C, von Bloh W (2006). Causes and timing of future biosphere extinctions. Biogeosciences.

[CR21] Franck S, Kossacki KJ, Bounama C (1999). Modelling the global carbon cycle for the past and future evolution of the earth system. Chemical Geology.

[CR22] Franck S, Kossacki KJ, Von Bloh W, Bounama C (2002). Long-term evolution of the global carbon cycle: Historic minimum of global surface temperature at present. Tellus B.

[CR23] Goldblatt C, Watson AJ (2012). The runaway greenhouse: Implications for future climate change, geoengineering and planetary atmospheres. Philosophical Transactions of the Royal Society A.

[CR24] Gough DO, Domingo V (1981). Solar interior structure and luminosity variations. Physics of solar variations.

[CR25] Häggstrom O (2016). Here be dragons: Science, technology and the future of humanity.

[CR26] Harrison G, Tanner J (2011). Better not to have children. Think.

[CR27] Hiller A, Gardiner SM, Thompson A (2017). Consequentialism in environmental ethics. The Oxford handbook of environmental ethics.

[CR28] Hiller A, Hiller A, Ilea R, Kahn L (2013). System consequentialism. Consequentialism and environmental ethics.

[CR29] Horta O (2011). The ethics of the ecology of fear against the nonspeciesist paradigm: A shift in the aims of intervention in nature. Between the Species.

[CR30] Horta O (2015). The problem of evil in nature: Evolutionary bases of the prevalence of disvalue. Relations beyond Anthropocentrism.

[CR31] Hudson HS (1991). A space parasol as a countermeasure against the greenhouse effect. Journal of the British Interplanetary Society.

[CR32] IPBES (2019). *Nature’s dangerous decline ‘unprecedented’; species extinction rates ‘accelerating’*. Intergovernmental Science-Policy Platform on Biodiversity and Ecosystem Services. Retrieved October 22, 2020, from https://ipbes.net/news/Media-Release-Global-Assessment.

[CR33] Johannsen K (2020). To assist or not to assist? Assessing the potential moral costs of humanitarian intervention in nature. Environmental Values.

[CR34] Kasting JF (1988). Runaway and moist greenhouse atmospheres and the evolution of Earth and Venus. Icarus.

[CR35] Katz E (1996). The problem of ecological restoration. Environmental Ethics.

[CR36] Kopparapu RK, Ramirez R, Kasting JF, Eymet V, Robinson TD, Mahadevan S, Terrien RC, Domagal-Goldman S, Meadows V, Deshpande R (2013). Habitable zones around main-sequence stars: New estimates. The Astrophysical Journal.

[CR37] Korycansky DG, Laughlin G, Adams FC (2001). Astronomical engineering: A strategy for modifying planetary orbits. Astrophysics and Space Science.

[CR38] Laskar J, Gastineau M (2009). Existence of collisional trajectories of Mercury, Mars and Venus with the Earth. Nature.

[CR39] Leclerc G-L (2018). The epochs of nature.

[CR40] Leconte J, Forget F, Charnay B, Wordsworth R, Pottier A (2013). Increased insolation threshold for runaway greenhouse processes on Earth-like planets. Nature.

[CR41] Lenton TM, von Bloh W (2001). Biotic feedback extends the life span of the biosphere. Geophysical Research Letters.

[CR42] Li K-F, Pahlevan K, Kirschvink JL, Yung YL (2009). Atmospheric pressure as a natural climate regulator for a terrestrial planet with a biosphere. Proceedings of the National Academy of Sciences of the United States of America.

[CR43] Liberg, O. (2005). Genetic aspects of viability in small wolf populations: With special emphasis on the Scandinavian wolf population. Report from an International Expert Workshop at Färna Herrgaard, Sweden, 1–3 May 2002. Swedish Environmental Protection Agency.

[CR44] Lovelock JE, Whitfield M (1982). Life span of the biosphere. Nature.

[CR45] Maher TM, Baum SD (2013). Adaptation to and recovery from global catastrophe. Sustainability.

[CR46] MacCormack P (2020). The Ahuman Manifesto: Activism for the end of the anthropocene.

[CR47] Malmberg D, Davies MB, Heggie DC (2011). The effects of fly-bys on planetary systems. Monthly Notices of the Royal Astronomical Society.

[CR48] Margulis L, Sagan D (1997). Microcosmos: Four billion years of microbial evolution.

[CR49] Martin JB (2017). Carbonate minerals in the global carbon cycle. Chemical Geology.

[CR50] Mautner MN (1991). A space-based solar screen against climatic warming. Journal of the British Interplanetary Society.

[CR51] McCumber A, King Z (2020). The wild in fire: Human aid to wildlife in the disasters of the anthropocene. Environmental Values.

[CR52] McInnes CR (2010). Space-based geoengineering: Challenges and requirements. Proceedings of the Institution of Mechanical Engineers Part C.

[CR53] McInnes CR, Badescu V, Cathcart RB, Schuiling RD (2006). Planetary macro-engineering using orbiting solar reflectors. Macro-engineering.

[CR54] McInnes CR (2002). Astronomical engineering revisited: Planetary orbit modification using solar radiation pressure. Astrophysics and Space Science.

[CR55] Moen OM (2016). The ethics of wild animal suffering. Etikk i Praksis (nordic Journal of Applied Ethics).

[CR56] Morton T (2007). Ecology without nature: Rethinking environmental aesthetics.

[CR57] Mill, J. S. (2002). *Utilitarianism* (Ed. Sher, G.). Hackett Publishing Co, Inc.

[CR58] Naess A (1973). The shallow and the deep, long-range ecology movement. A summary. Inquiry.

[CR59] Newbold T (2018). Future effects of climate and land-use change on terrestrial vertebrate community diversity under different scenarios. Proceedings of the Royal Society B.

[CR60] Nordhaus WD (1997). discounting in economics and climate change; an editorial comment. Climatic Change.

[CR61] O’Malley-James JT, Greaves JS, Raven JA, Cockell CS (2012). Life and light: Exotic photosynthesis in binary and multiple-star systems. Astrobiology.

[CR62] Ord T (2020). The precipice: Existential risk and the future of humanity.

[CR63] Owe, A. (2022). Greening the universe: The case for ecocentric space expansion. In J. S. J. Schwartz, L. Billings, & E. Nesvold (Eds.), *Forthcoming in reclaiming space: Progressive and multicultural visions of space exploration*. James Oxford University Press.

[CR64] Ozaki K, Reinhard CT (2021). The future lifespan of Earth’s oxygenated atmosphere. Nature Geoscience.

[CR66] Parfit D (1984). Reasons and persons.

[CR67] Pearson J, Oldson J, Levin E (2006). Earth rings for planetary environment control. Acta Astronautica.

[CR68] Persson I, Savulescu J (2008). The perils of cognitive enhancement and the urgent imperative to enhance the moral character of humanity. Journal of Applied Philosophy.

[CR70] Pierrehumbert RT (2010). Principles of planetary climate.

[CR69] Rawls J (1999). A theory of justice.

[CR71] Rolston H (1992). Disvalues in nature. The Monist.

[CR72] Rolston H (1985). Duties to endangered species. BioScience.

[CR73] Rolston H (2012). A new environmental ethics. The next millennium for life on earth.

[CR74] Rushby, A. J. (2015). Modelling biogeochemical controls on planetary habitability. PhD Thesis, University of East Anglia.

[CR75] Rushby AJ, Claire MW, Osborn H, Watson AJ (2013). Habitable zone lifetimes of exoplanets around main sequence stars. Astrobiology.

[CR76] Sagan C, Mullen G (1972). Earth and Mars: Evolution of atmospheres and surface temperatures. Science.

[CR77] Sandler R (2010). The value of species and the ethical foundations of assisted colonization. Conservation Biology.

[CR78] Scanlon T (1998). What we owe to each other.

[CR79] Scheffler S (2018). Why worry about future generations?.

[CR80] Schopenhauer A (1958). The world as will and representation.

[CR81] Schröder K-P, Connon Smith R (2008). Distant future of the sun and earth revisited. Monthly Notices of the Royal Astronomical Society.

[CR82] Sloan D, Batista RA, Loeb A (2017). The resilience of life to astrophysical events. Scientific Reports.

[CR83] Snyder-Beattie AE, Sandberg A, Drexler KE, Bonsall MB (2021). The timing of evolutionary transitions suggests intelligent life is rare. Astrobiology.

[CR84] Taylor PW (1981). The ethics of respect for nature. Environmental Ethics.

[CR85] Teller, E., Wood, L., & Hyde, R. (1996) Global warming and ice ages: I. Prospects for physics-based modulation of global change. Lawrence Livermore National Laboratory. (UCRL-JC-128715). United States.

[CR86] Tonn BE (2002). Distant futures and the environment. Futures.

[CR87] Tonn BE (1999). Transcending oblivion. Futures.

[CR88] Toon OB (2014). Environmental consequences of nuclear war. AIP Conference Proceedings.

[CR65] Toon OB, Robock A, Turco RP (2014). Environmental consequences of nuclear war. AIP Conference Proceedings.

[CR89] von Bloh W, Franck S, Bounama C, Schellnhuber H-J (2003). Biogenic enhancement of weathering and the stability of the ecosphere. Geomicrobiology Journal.

[CR90] von Hartmann E (2014). Philosophy of the unconscious.

[CR91] Washington H, Taylor B, Kopnina H, Cryer P, Piccolo JJ (2017). Why ecocentrism is the key pathway to sustainability. The Ecological Citizen.

[CR92] Walker JC, Hays PB, Kasting JF (1981). A negative feedback mechanism for the long-term stabilization of earth’s surface temperature. Journal of Geophysical Research: Oceans.

[CR93] Wolf ET, Toon OB (2015). The evolution of habitable climates under the brightening Sun. Journal of Geophysical Research: Atmospheres.

[CR94] Wolf ET, Toon OB (2014). Delayed onset of runaway and moist greenhouse climates for earth. Geophysical Research Letters.

